# The PPAR pan-agonist bezafibrate ameliorates cardiomyopathy in a mouse model of Barth syndrome

**DOI:** 10.1186/s13023-017-0605-5

**Published:** 2017-03-09

**Authors:** Yan Huang, Corey Powers, Victoria Moore, Caitlin Schafer, Mindong Ren, Colin K. L. Phoon, Jeanne F. James, Alexander V. Glukhov, Sabzali Javadov, Frédéric M. Vaz, John L. Jefferies, Arnold W. Strauss, Zaza Khuchua

**Affiliations:** 1 The Heart Institute, Department of Pediatrics, the University of Cincinnati College of Medicine and Cincinnati Children’s Hospital Medical Center, 240 Albert Sabin Way, Cincinnati, OH 45229-7020 USA; 2 Departments of Anesthesiology and Cell Biology, New York University School of Medicine, New York, NY USA; 3 Department of Pediatrics, New York University School of Medicine, New York, NY USA; 4 Department of Biochemistry, I.M. Sechenov First Moscow State Medical University, Moscow, Russian Federation; 5 Department of Physiology, University of Puerto Rico School of Medicine, San Juan, Puerto Rico; 6 Academic Medical Center, Department of Clinical Chemistry and Pediatrics, Laboratory of Genetic Metabolic Disease (F0-224), Meibergdreef 9, 1105 AZ Amsterdam, The Netherlands

**Keywords:** Barth syndrome, Cardiolipin, Mitochondria, Cardiomyopathy, Systolic dysfunction, Fibrates

## Abstract

**Background:**

The PGC-1α/PPAR axis has been proposed as a potential therapeutic target for several metabolic disorders. The aim was to evaluate the efficacy of the pan-PPAR agonist, bezafibrate, in tafazzin knockdown mice (TazKD), a mouse model of Barth syndrome that exhibits age-dependent dilated cardiomyopathy with left ventricular (LV) dysfunction.

**Results:**

The effect of bezafibrate on cardiac function was evaluated by echocardiography in TazKD mice with or without beta-adrenergic stress. Adrenergic stress by chronic isoproterenol infusion exacerbates the cardiac phenotype in TazKD mice, significantly depressing LV systolic function by 4.5 months of age. Bezafibrate intake over 2 months substantially ameliorates the development of LV systolic dysfunction in isoproterenol-stressed TazKD mice. Without beta-adrenergic stress, TazKD mice develop dilated cardiomyopathy by 7 months of age. Prolonged treatment with suprapharmacological dose of bezafibrate (0.5% in rodent diet) over a 4-month period effectively prevented LV dilation in mice isoproterenol treatment. Bezafibrate increased mitochondrial biogenesis, however also promoted oxidative stress in cardiomyocytes. Surprisingly, improvement of systolic function in bezafibrate-treated mice was accompanied with simultaneous reduction of cardiolipin content and increase of monolysocardiolipin levels in cardiac muscle.

**Conclusions:**

Thus, we demonstrate that bezafibrate has a potent therapeutic effect on preventing cardiac dysfunction in a mouse model of Barth syndrome with obvious implications for treating the human disease. Additional studies are needed to assess the potential benefits of PPAR agonists in humans with Barth syndrome.

## Background

Barth syndrome (BTHS) is a rare genetic disorder that affects multiple organs. The mutated gene causing BTHS is *(TAZ)*, which encodes a mitochondrial transacylase tafazzin, a key enzyme in the cardiolipin (CL) remodeling pathway. Mutations in the *TAZ* result in CL deficiency, and increase the monolysocardiolipin (MLCL) to cardiolipin ratio (MLCL/CL) accompanied by structural and functional defects in mitochondria of affected individuals and *Taz* knockdown (TazKD) mice [1, 2]. This mitochondrial CL deficiency destabilizes the integrity and activity of electron transfer chain (ETC.) complexes [3–5]. The human phenotype includes dilated cardiomyopathy, underdeveloped skeletal musculature, and intermittent neutropenia [6]. Inactivation of *Taz* with doxycycline-inducible (tet-on) shRNA-mediated gene silencing in mice results in the LV dilation and systolic dysfunction after 6 months of age [1, 7]. In addition, previous studies also reported the embryonic lethality in TazKD mice when knockdown was initiated with doxycycline at high dose [8].

Current therapies for BTHS are limited and have variable efficacy. These include antioxidants, a diet supplemented with specific amino acids, and granulocyte colony stimulating factor (GCSF) to treat neutropenia. Patients with BTHS may develop progressive heart failure and require mechanical circulatory support and cardiac transplantation [9]. An acute decline in health from a stable status to a life-threatening crisis can occur with little warning so that patients and their families live in a constant state of anxiety. Developing effective therapies for BTHS continues to be a challenge, especially because of the limited number of patients, extraordinary phenotypic variability, and unpredictable clinical course [9–11].

Energy limitation plays a major role in heart failure [12]. The replenishment of energy supply in cardiac cells by metabolic therapy is an expanding opportunity in the treatment of heart failure. Due to a central role in energy metabolism and mitochondrial bioenergetics, peroxisome proliferator-activated receptors (PPARs) may be potential therapeutic targets for metabolic targeted therapy to ameliorate cardiac dysfunction induced by *Taz* deficiency. Indeed, beneficial effects of activation of the PPAR/PGC1α axis have been demonstrated in various mitochondrial disorders. Pharmacological activation of PPARα facilitates post-ischemic functional recovery in hypertrophied neonatal rabbit hearts [13] and slows down the progression of the left ventricular dysfunction in the porcine model of tachycardia-induced cardiomyopathy [14]. In patients with metabolic syndrome, BF reduces the incidence of myocardial infarction and lowers cardiac mortality risk [15]. Treatment with BF provided beneficial effects in patients with carnitine palmitoyltransferase-II (CPT-II) deficiency [16], although other studies reported conflicting results [17, 18]. Here, we report the results of experimental treatment with BF in a mouse model of BTHS, with an shRNA-mediated knockdown of *Taz* expression (TazKD) that exhibits age-dependent cardiomyopathy [1, 7].

## Methods

### Animals

All animal studies were approved by the Institutional Animal Care and Use Committee of Cincinnati Children’s Hospital Medical Center. Animals were housed in micro-isolator cages at 25^o^ C under a 14/10 h light/dark cycle with free access to drinking water and food. Doxycycline-inducible shRNA-mediated TazKD mice have been described previously [1, 4, 7]. *Taz* knockdown was induced prior to conception by feeding females doxycycline-containing rodent chow (625 mg/kg) 3 days before mating. This approach allows 85–95% silencing of *Taz* in heart and skeletal muscle [1]. In the ensuing offspring, after 3 months of age, the doxycycline administration route was switched to drinking water (0.1% doxycycline, 10% sugar). Both WT and TazKD mice were continuously maintained on doxycycline-containing water for the duration of the study. Male mice with C57BL/6 background were used in experiments.

Micro-osmotic pumps (Alzet model 1002) were used to deliver controlled amounts (0.25 μL/h) of isoproterenol (Iso), a β-adrenergic agonist, to mice for 14 days. Pumps were loaded with a dose of Iso consistent with 30 mg/kg/day. All aseptic procedures were performed in a rodent surgical suite. Mice were anesthetized with 1.75% isoflurane. A small incision of approximately 3–5 mm was made at the nape of the neck. The pump was inserted and CV-22 biodegradable suture was used to close the wound. Then the mice were returned to their original cages for recovery.

At 3 months of age, mice were given specifically formulated pelleted rodent chow that contained either no or 0.5% bezafibrate (TestDiet, St. Louis, MO) provided at libitum for 2 or 4 months. At the same time, the doxycycline administration route was changed for all animals from rodent chow to drinking water with 0.1% of doxycycline and 10% sucrose since manufacturing and sterilizing the rodent chow that contained both bezafibrate and doxycycline was technically difficult.

### Echocardiography

Two-dimensional and M-mode transthoracic echocardiography were performed under isoflurane anesthesia as previously described using a Vevo 2100 Micro-Imaging system (VisualSonics, Inc.) and a 40 MHz transducer [1]. Off-line analyses were performed by investigators blinded to genotype.

### Quantitative PCR

Quantitative PCR- based assay was used to determine mitochondrial DNA (mtDNA) copy numbers relative to the diploid chromosomal DNA content. Fragment of mtDNA was amplified using ACTATCCCCTTCCCCATTTG and GCTACCCCCAAGTTTAATGG primer pair. Fragment of chromosomal DNA was amplified with ACAAAGCAAAGGAGCTGGAG and TCATTGCCACTGCTGAGAAC primers. *Taz* expression was analyzed with quantitative RT-PCR using murine *Taz*-specific primers ATGCCCCTCCATGTGAAGTG and TGGTTGGAGACGGTGATAAGG. Results are expressed relative to β-actin mRNA content, that was determined using following primer set: AAGAGCTATGAGCTGCCTGA and ACGGATGTCAACGTCACACT. Quantitative PCR was performed using a Realplex Mastercycler (Eppendorf) and SYBR Green RT-PCR reagent (Bio-Rad). PCR conditions were 30 s at 95 °C, 30 s at 55 °C, and 30 s at 68 °C for 35 cycles.

### Western blot analysis

Western immunoblot analyses were done by standard techniques using NuPAGE Novex Bis-Tris pre-cast gels (Life Technologies, Carlsbad, CA). Tissues were homogenized in ice-cold buffer composed of 0.1 M KH_2_PO_4_, 2 mM EDTA, 2% Triton X-100 and protease inhibitor cocktail (Roche, Basel, Switzerland). Homogenates were centrifuged at 12 000 × g at 4o C and protein concentrations in supernatants were determined with the BCA assay (BioRad, Hercules, CA). After electrophoresis, proteins were transferred to nitrocellulose membranes. Membranes were blocked with 5% BSA overnight. Protein levels of ETC. complexes were detected with a cocktail of mouse monoclonal antibodies specific to selected subunits of the complexes (Life Technologies). Custom-made antibodies specific to mitochondrial malate dehydrogenase (mMDH) were used for the loading control. Secondary IRDye antibodies were used for imaging (Licor Biosciences, Lincoln, NE). Membranes were scanned with an Odyssey CLx scanner. Band intensity analysis and data quantification were done with Image Studio software.

### Cardiolipin analysis

Tissue homogenates were made in Milli-Q water in a Qiagen Tissuelyser II using stainless steel beads of 5 mm for two times 30 s at 30 rev/s. The protein concentration of the homogenates was determined with the BCA assay. Phospholipids were extracted using a single-phase extraction. An internal standard (CL(14:0)_4_ was added to all samples containing 1 mg of protein followed by 1.5 mL of chloroform/methanol (1:1, v/v). Subsequently, the mixtures were sonicated in the water bath for 5 min, followed by centrifugation at 16,000 × g for 5 min. The supernatants (organic layer) were then transferred to the glass vials and evaporated under a nitrogen stream at 45 °C. Subsequently, the residues were dissolved in 150 μL of chloroform/methanol (9:1, v/v), and 10 μL of the solution was injected into the HPLC-MS system.

The HPLC system consisted of an Ultimate 3000 binary (U) HPLC pump, a vacuum degasser, a column temperature controller, and an autosampler (Thermo Scientific, Waltham, MA, USA). The column temperature was maintained at 25 °C. The lipid extracts were injected onto a LiChrospher 2* 250-mm silica-60 column, 5 μm particle diameter (Merck, Darmstadt, Germany). The phospholipids were separated from interfering compounds by a linear gradient between solution B (chloroform/methanol, 97:3, v/v) and solution A (methanol/water, 85:15, v/v). Solutions A and B contained 5 and 0.2 ml of 25% (v/v) aqueous ammonia per liter of eluent, respectively. The gradient (0.3 ml/min) was as follows: 0–1 min 10%A, 1–4 min, 10%A–20%A, 4–12 min 20%A–85% A; 12–12.1 min, 85%A–100% A; 12.1–14.0 min, 100% A, 14–14.1 min, 100%A–10%A and 14.1–15 min, equilibration with 10% A. All gradient steps were linear, and the total analysis time, including the equilibration, was 15 min. A Q Exactive Plus MS (Thermo Scientific) was used in the negative and positive electrospray ionization mode. Nitrogen was used as the nebulizing gas. The source collision-induced dissociation collision energy was set at 0 V. The spray voltage was 2500 V, and the capillary temperature was 256 °C. In both negative and positive modes, mass spectra of phospholipid molecular species were obtained by continuous scanning from m/z 150 to m/z 2000 with a resolution of 280.000 (FMWH at m/z 200).

For bioinformatic analysis of the data, the raw HPLC/MS data were converted to an mzXML format using msConvert for the Negative Scan data and ReAdW for the Positive Scan data. The data set was processed using a semi-automated metabolomics pipeline written in the R programming language [http://www.r-project.org]. In brief, it consisted of the following five steps: (1) pre-processing, (2) identification of metabolites, (3) isotope correction, (4) normalization and scaling and (5) statistical analysis, using the XCMS R package.

### Isolation of mitochondria and analysis of ETC complex I-III activity

Cardiac mitochondria were isolated and enzymatic activities of complex I-III segment and citrate synthase were measured spectrophotometrically, as previously described [4].

### Statistical analysis

Statistical analysis, reported as means ± standard deviations, was performed with one-way ANOVA. Post-hoc analyses of echocardiographic indices were performed using the Mann-Whitney non-parametric test. For statistical comparison of cardiolipin content between the groups, one-way ANOVA with post-hoc Bonferroni correction was used. A probability value of 0.05 or lower was considered significant.

## Results

### Chronic isoproterenol treatment exacerbates the cardiac dysfunction in TazKD mice

**Fig. 1:**
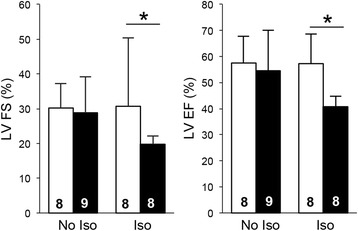
Isoproterenol (iso) exacerbates systolic dysfunction in TazKD mice. Fractional shortening (FS) and ejection fraction (EF) indices in 4.5-5 month-old isoflurane-sedated WT and TazKD mice (*open and black bars*, respectively). Numbers in the *bars* represent the sample size for the corresponding data. Data are presented as means ± standard deviation. Asterisks (*) depict statistical significance (*p* < 0.05) between groups

Without stress, 4.5-month-old TazKD mice exhibited normal systolic function and did not differ from WT controls. We investigated whether chronic cardiac stress secondary to β-adrenergic stimulation would aggravate the cardiac phenotype in TazKD mice. Isoproterenol was administered to 2.5-month-old WT and TazKD mice using subcutaneously implanted micro-osmotic pumps. Echocardiographic assessment of cardiac function in Iso-treated mice was performed at 4.5 months of age. Results show that systolic indices were preserved in Iso-treated WT mice (Fig. 1). In contrast, in TazKD mice, treatment with Iso for 2 weeks unveiled significant impairment in cardiac contractile function. Left ventricular ejection fraction (LV EF) and left ventricular fractional shortening (LV FS) were markedly reduced in Iso-treated TazKD mice compared to untreated TazKD mice or Iso-treated WT controls, consistent with impending cardiomyopathy (Fig. 1). Thus, TazKD in mice had no obvious impact on systolic function at 4.5 months of age. At the same age, TazKD mice undergoing an additional β-adrenergic stress developed a more severe cardiac phenotype with impaired systolic function.

### Bezafibrate ameliorates isoproterenol-induced heart failure in TazKD mice

We investigated whether the PPAR agonist, BF might ameliorate cardiomyopathy and improve cardiac function in Iso-treated TazKD mice. Cohorts of 4.5-month-old WT and TazKD mice with implanted Iso-delivering micro-osmotic pumps were given rodent chow that contained 0.5% BF. At the same time, the doxycycline administration route was changed for all animals from rodent chow to drinking water with 0.1% of doxycycline and 10% sucrose because manufacturing and sterilizing the rodent chow that contained both BF and doxycycline was technically difficult.

**Fig. 2:**
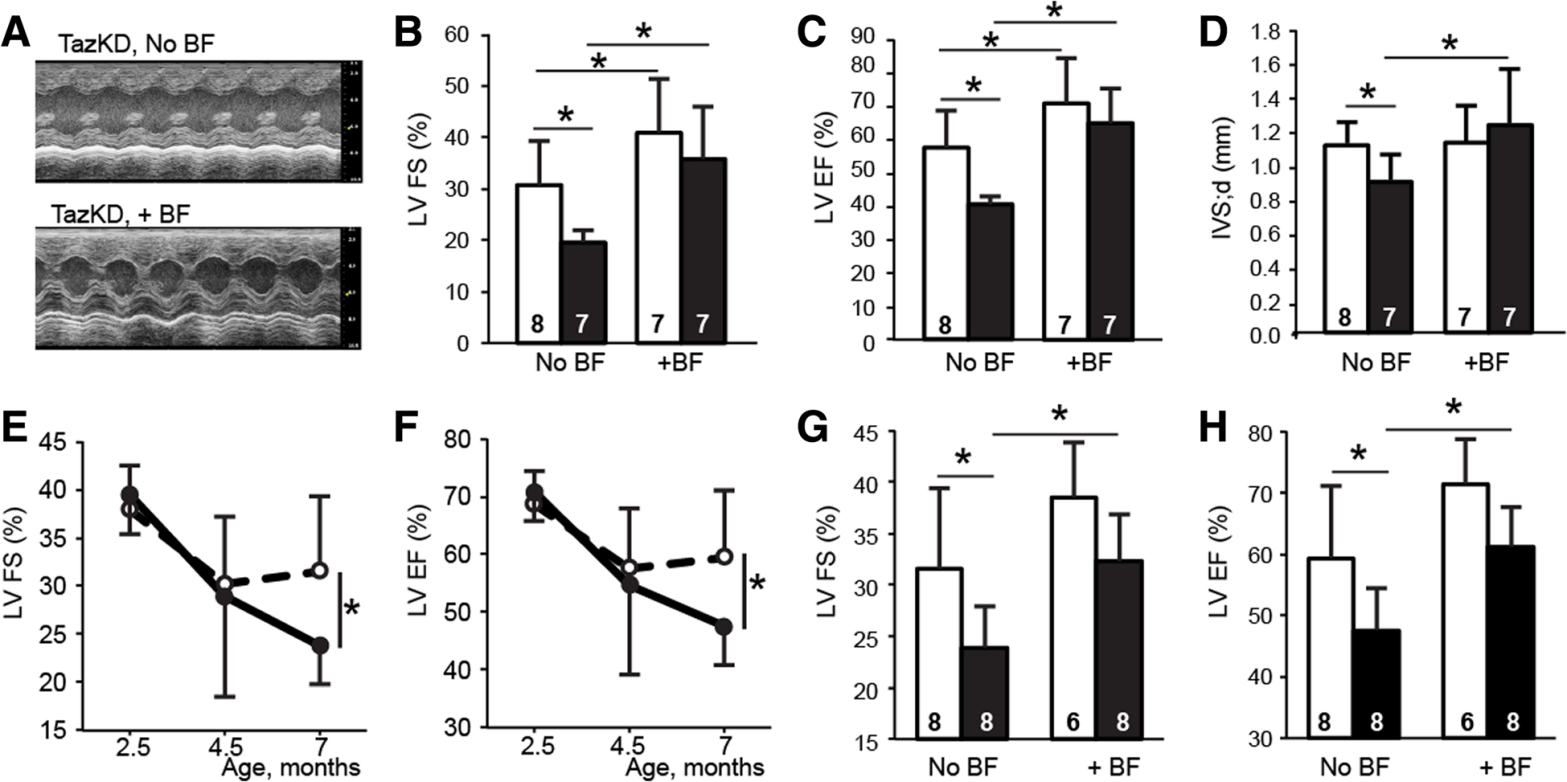
Bezafibrate (BF) ameliorates systolic function in TazKD mice. **a** Representative M-mode echocardiographic recordings of 4.5 months old iso-treated TazKD mice fed a diet with or without BF. **b**–**d** Effects of BF on (**b**) LV fractional shortening (LV FS), (**c**) ejection fraction (LV EF) and (**d**) end-diastolic interventricular wall thicknesses (IVS;d) in 4.5-month-old iso-treated WT (*open bars*) and TazKD (*black bars*) mice. **e** and **f** LV FS and LV EF indices are shown for 2.5, 4.5 and 7 months old WT (*dashed line*) and TazKD (*solid line*) mice. **g** and **h** Effects of BF on LV FS and LV EF in 7 months old WT (*open bars*) and TazKD mice (*black bars*). Mice were maintained on a diet with or without 0.5% BF during the 2 months between 2.5 and 4.5 months of age (**a**-**d**), or 4 months between 3 and 7 months of age (**g** and **h**). Numbers in the *bars* represent the sample size for the corresponding data. Data are presented as means ± standard deviation. Asterisks (*) depict statistical significance (*p* < 0.05) between groups

After 2 months on the BF-containing diet, cardiac function in mice was examined by echocardiography. In WT mice BF increased LV FS from 31 ± 9% in the untreated group to 41 ± 10% (*P <* 0.05). LV EF was improved from 57 ± 11% in the untreated group to 71 ± 13% in the BF-treated group (*P <* 0.05) (Fig. 2b,c).

BF significantly improved cardiac function in Iso-treated TazKD group. LV FS showed a dramatic increase from 20 ± 2% in the untreated group to 36 ± 10% in the BF-treated group (*P <* 0.001). LV EF was improved from 40 ± 2 in the untreated group to 65 ± 10% in the BF-treated group (*P <* 0.001) (Fig. 2b,c). *TAZ* knockdown in Iso-treated model resulted in reduction of end-systolic thickness of interventricular septum (IVS;d) from 1.10 ± 0.12 mm in WT group to 0.88 ± 0.15 mm in TazKD group (*P <* 0.01). BF increased thickness of IVS;d in TazKD mice to 1.21 ± 0.31 mm (*P <* 0.05), however, had no impact on IVS;d in WT mice (Fig. 2d).

### Bezafibrate treatment improves systolic function in 7-month-old TazKD mice

Next, we investigated the effects of extended BF treatment on WT and TazKD mice without β-adrenergic stress. Cohorts of WT and TazKD mice were subjected to echocardiographic evaluation of cardiac function at 2.5, 4.5 and 7 months of age. TazKD mice with no β-adrenergic stress exhibited normal contractile function at age of 4.5 months, and systolic defects became apparent by age of 7 months, consistent with previously reported studies in this mouse model [1] (Fig. 2e,f). TazKD mice at 7 months of age demonstrated signs of cardiac dysfunction with impaired LV FS (32 ± 8 and 24 ± 4% for WT and TazKD, respectively; *P <* 0.05), and LV EF (59 ± 12 and 48 ± 7% for WT and TazKD, respectively; *P <* 0.05).

Treatment with BF, starting from 3 months of age, prevented the development of cardiomyopathy in 7-month-old TazKD mice by preserving LV FS and EF indices. BF increased LV FS values from 24 ± 4% for untreated group to 32 ± 5% for BF-treated group (*P <* 0.001) (Fig. 2g). Similarly, LV EF values were 48 ± 7 and 61 ± 6% for untreated and BF-treated TazKD groups, respectively (*P <* 0.001) (Fig. 2h). Prolonged treatment with BF demonstrated a tendency of increasing LV FS and LV EF values in 7-month-old WT mice as well, although the difference did not quite reach statistical significance.

### Bezafibrate treatment reduces cardiolipin content and alters its molecular speciation in cardiac muscle

**Fig. 3:**
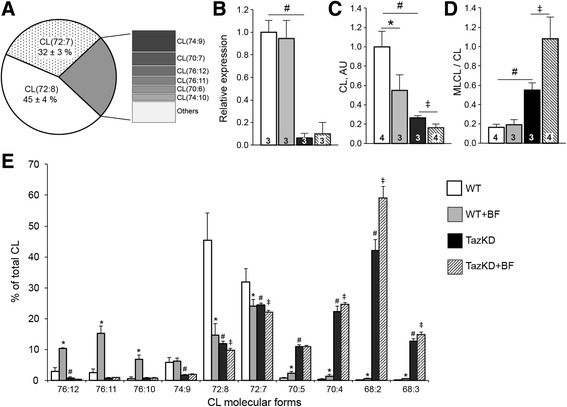
Analysis of cardiolipin (CL) molecular species in hearts of 7-month-old WT and TazKD mice fed a diet with or without 0.5% BF. **a** Molecular forms of CL in 7 months old WT mouse heart. Relative distribution of molecular forms of CL as a percentage of total CL content is shown. **b** Relative expression of Taz in cardiac muscles of WT and TazKD mice with and without BF treatment. **c** Changes of absolute values of CL in cardiac muscles of WT and TazKD mice with and without BF treatment. **d** Monolysocardiolipin (MLCL) to CL ratios in cardiac muscles of WT and TazKD mice with and without BF treatment. Numbers in the *bars* on panels **b**-**d** represent the sample size for the corresponding data. **e** Relative changes of major molecular forms of CL in WT and TazKD mice with and without BF treatment. Data are presented as means ± standard deviation. Sample sizes are shown. Statistically significant differences (*p* < 0.05) are indicated: *, between untreated and BF-treated WT mice; #, between WT and TazKD mice without BF treatment; ‡, between untreated and BF-treated TazKD mice

In the next set of experiments, we analyzed impacts of BF on content and molecular spectrum of CL species in hearts of 7-month-old WT and TazKD mice. Cardiolipin profiling revealed that molecular forms of CL with four C18 acyl groups, corresponding to CL(72:7) and CL(72:8) species, constitute approximately 80% of the total CL in WT hearts (Fig. 3a). This is consistent with previous reports [1, 19]. Doxycycline-inducible shRNA-mediated gene knockdown reduced *Taz* mRNA level in cardiac muscle of TazKD mice by 95%. BF had no impact on *Taz* expression in cardiac muscle either in WT or in TazKD mice (Fig. 3b). As expected, *Taz* knockdown reduced the content of total CL and increased MLCL to CL ratio in hearts TazKD mice (Fig. 3c, d). Surprisingly, treatment with BF significantly reduced content of total CL and increased MLCL/CL ratios both in WT and TazKD mice. We further investigated impact of BF on CL molecular speciation in WT and TazKD mouse hearts. In hearts of WT mice BF reduced content of CL(72:7) and CL(72:8), and increased a content of CLs with longer and more unsaturated acyl groups (Fig. 3e). However, in TazKD hearts BF increased the levels of CLs with shorter and more saturated acyl groups such as CL(70:4), CL(68:2) and CL(68:3) (Fig. 3e).

### BF promotes mitochondrial biogenesis in cardiomyocytes

**Fig. 4:**
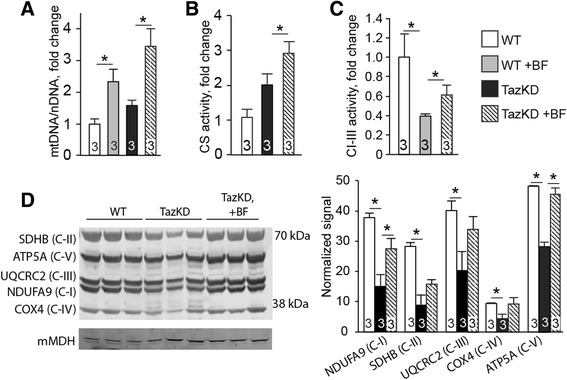
Effects of BF on mitochondria. **a** Mitochondrial DNA (mtDNA) copy numbers were analyzed with qPCR of total DNA from cardiac tissues and normalized to nuclear DNA (nDNA) content. **b** Citrate synthase (CS) activities were measured in cardiac homogenates and normalized to protein concentrations. Data are depicted as fold changes relative to WT controls. **c** Enzymatic activity of RC segment I-III in mitochondria from 7 month-old WT and TazKD mice with and without BF treatment. Values are normalized to CS activity and are shown as fold-changes relative to WT controls. **d** Western blot. 20 μg protein samples from 7 month-old untreated WT, untreated TazKD, and BF-treated TazKD mouse hearts were analyzed. A cocktail of monoclonal antibodies specific to selected polypeptides of the RC complexes were used. Signal intensities were normalized relative to mitochondrial malate dehydrogenase (mMDH) and plotted for each marker

Quantitative PCR analysis of total DNA from mouse hearts revealed an approximately 60% (*P* < 0.05) increase of mtDNA content in the hearts of 7-month-old untreated TazKD mice compared to untreated WT counterparts (Fig. 4a). Bezafibrate further increased mtDNA content in LV muscle of TazKD mice approximately by 200% (*P* < 0.01). We also observed a similar increase in mitochondrial citrate synthase activity in BF-treated hearts, suggesting that BF enhances mitochondrial biogenesis in TazKD hearts (Fig. 4b). We have previously reported that TazKD mice have impaired activity of mitochondrial ETC. complexes I–III [4]. Bezafibrate increased enzymatic activity of the segment I-III of ETC. complexes in cardiac mitochondria by 35% (*P* < 0.05) (Fig. 4c). Impact of BF on mitochondrial ETC. complexes was further evaluated in protein extracts from LV muscles by semi-quantitative western blot analysis using the cocktail of antibodies specific to selected subunits of ETC. complexes. Cardiac mitochondria isolated from TazKD mice exhibited significantly reduced content of all tested subunits of ETC. complexes. Treatment with BF during 4 months showed tendency to increase protein expression of all ETC. complexes, although the differences did not reach statistical significance, except for NDUFA9 (complex I) (Fig. 4d).

## Discussion

In humans with mutations in energy metabolism genes, physiological stressors, such as fasting, cold exposure, exercise, or infections can rapidly induce life-threatening events in otherwise stable and asymptomatic patients [20–22]. This is certainly the situation in BTHS. Similarly, mouse models of energy metabolism disorders often require additional physiological stressors, e.g. fasting, cold-exposure, β-adrenergic stimulation or a high-fat diet, to evoke a cardiac phenotype [23–26]. Isoproterenol, a β-adrenergic agonist, acts as a specific pathological stressor to exacerbate cardiac phenotypes and has become a useful tool to study cardiac disorders in animal models [27].

Cardiomyopathy in humans with BTHS may present earlier; prenatally or early postnatally. In mice, despite the same genotype, two distinct phenotypes have been reported. Prior reports of this model demonstrated that cardiomyopathy develops only in adult mice, when knockdown is induced with relatively low dose of doxycycline (25–80 mg/kg/daily) [1, 7]. In contrast, fetal cardiac defects and embryonic or early postnatal lethality were observed when Taz knockdown was initiated *in utero* with higher dose of doxycycline (~300 mg/kg/daily) [8]. A plausible cause of distinct phenotypes of these two models with the same genotype is the dose of doxycycline. Seibler et al. showed that the dose of doxycycline influenced the speed with which the knockdown achieved a steady-state level [8, 28].

In this study, we show that β-adrenergic stimulation resulted in LV systolic dysfunction in TazKD mice at 4.5 months of age, while in those not receiving a β-adrenergic challenge, the cardiac phenotype becomes apparent after 7 months of age. These results suggest that Taz deficiency in adult mice is better compensated than in humans and additional external stressors are necessary to elicit the cardiac phenotype.

Bezafibrate is a pan-activator of PPAR signaling and promotes transcriptional activation of genes involved in oxidative metabolism [29]. BF activates PPAR-PGC1α signaling and mitochondrial biogenesis in brain, ameliorating Huntington’s disease, mitochondrial encephalopathy and tau pathology phenotypes in mice [30–32]. BF improves substrate metabolism and reduces right ventricular hypertrophy in congestive heart failure model [33]. Fibrates slow down the progression of the LV dysfunction in tachycardia-induced cardiomyopathy [14]. Interestingly, overexpression of PGC1α induced mitochondrial biogenesis in the skeletal muscles of cytochrome c oxidase (complex IV) deficient mice. However, BF failed to induce mitochondrial biogenesis and even showed adverse effects on the skeletal muscles of mice with various mitochondrial myopathies [34–36]. Published reports suggest that BF may evoke distinct responses in different tissues: BF rescues mitochondrial defects in liver, skin, spleen and heart inducing mitochondrial biogenesis in these tissues [13, 16, 36]. However, BF has no beneficial effects, or even could be detrimental to glycolytic skeletal muscles [17, 34, 37].

BF is commonly prescribed to patients with dyslipidemia and diabetes. In humans, BF is used at 10 mg/kg daily dose. At this dosage, BF is undetectable in plasma and reportedly ineffective for PPAR activation in rodents. The pharmacological effect on lipogenesis is achieved by transcriptional down-regulation of sterol regulatory element-binding protein 1c in liver tissue via a PPAR-independent mechanism. Low-dose BF (10 mg/kg/day) failed to have any significant impact on the expression of PPARα, PGC1α and fatty acid oxidation genes in the liver [29]. In rodents, BF is commonly administered via a chow diet containing 0.5% of the drug that corresponds to 600–800 mg/kg/daily dose. This dose is approximately 60–80 times higher than the usual dose for humans for the treatment of dyslipidemia [30, 31, 34–36, 38]. At dosage of 0.5%, BF is well tolerated by rodents. These observations prompted us to use BF in the same dosage.

BF effectively ameliorated cardiac phenotypes in Iso-treated TazKD mice. These experiments strongly suggest that the PPAR/PGC1α signaling system is a promising therapeutic target for cardiomyopathy in patients with BTHS. However, Iso evokes complex responses in cardiomyocytes at high dosage (100 mg/kg) and may be toxic to cells, promoting oxidative stress and apoptosis [39]. We wanted to show that the observed therapeutic effect of BF on Iso-pretreated TazKD mice was not mediated by the protection of the heart from β-adrenergic stress. Thus, in the separate set of experiments, we administered BF to mice without Iso pretreatment. In this series, we administered BF for a prolonged period between 3 and 7 months of age. BF markedly improved contractile function in TazKD mice at 7 months of age, showing that therapeutic action of BF is not mediated by the protection of the heart from β-adrenergic stress.

Taz knockdown caused a significant increase of mtDNA content and showed a tendency of increased citrate synthase activity in cardiomyocytes. These observations are indicative of adaptive mitochondrial proliferation in TazKD cardiac muscle in response to energy deficiency and are consistent with previous observations [1]. However, mitochondrial proliferation in hearts of untreated TazKD mice was accompanied by reduction of electron flow through CI-III segment of ETC. and diminished content of several subunits of ETC. complexes. These observations are consistent with previously reported proteomic studies on TazKD mice [3]. BF further increased mitochondrial DNA content, citrate synthase activity, partially augmented electron flow through the CI-CIII segment of ETC. and demonstrated a tendency to increase expression of ETC. proteins in TazKD mice. Interestingly, an increase of the CI-III activity occurred despite the deterioration of CL content in the mitochondria. It is plausible that an increased turnover rates of individual subunits or chaperones of ETC. complexes I and III compensate for enzyme deficiency, even if CL is depleted in the mitochondria.

Although BF had no effect on *Taz* expression, it caused notable changes in molecular speciation of cardiolipin. BF significantly reduced content of tetralinoleoyl CL and increased content of MLCLs in cardiac muscle. In WT group, BF increased content of minor CL species with (76:12) – (76:10) acyl groups corresponding to C19 odd chain polyunsaturated fatty acids (Fig. 3e). However, in TazKD group, there was an opposite shift towards to CL species with shorter and less-saturated side chains. The physiological significance of these shifts is unclear.

## Conclusion

In summary, the principal findings of this study are: 1) Iso exacerbates the cardiac phenotype in TazKD mice rapidly by inducing dilated cardiomyopathy and LV systolic dysfunction by 4.5 months of age, and 2) the pan-PPAR agonist BF effectively prevents development of LV dilation and LV systolic dysfunction in TazKD mice. The study raised some points that require further investigation. First, cardiac function of TazKD mice improves in response to BF treatment despite deterioration of CL, a classical biomarker of BTHS. This striking outcome is likely to be caused by increased mitochondrial biogenesis in BF-treated hearts without parallel upregulation of tafazzin or other CL-remodeling enzymes. To our knowledge, this is a first experimental observation of improving cardiac function in the settings of diminished cellular levels of CL. Precise mechanisms underlying this improvement are unclear. Our results suggest that BF-induced biogenesis of more CL-depleted and therefore, less efficient mitochondria, are sufficient to reverse an adverse cardiac phenotype and improve heart contractility in TazKD mice. Second, bezafibrate dosage in our mouse model is 60–80 times higher than the therapeutic dose for humans. BF is well tolerated in humans. Subjective side-effects from the gastrointestinal tract (abdominal pain and nausea) occur in some patients that received equivalent of 17–20 mg/kg daily dose [40]. However, use of a higher dose of BF in potential human trials would be challenging. Therefore, a minimal effective dose should be experimentally determined first in the animal model with organ-level pharmacokinetic analysis, conservative assessment of end-organ toxicity and L4CL/MLCL levels.

## Abbreviations

BF: Bezafibrate
BTHS: Barth syndrome
PPAR: Peroxisome proliferator-activated receptor

## References

[1] Acehan D, Vaz F, Houtkooper RH, James J, Moore V, Tokunaga C, Kulik W, Wansapura J, Toth MJ, Strauss A (2011). Cardiac and skeletal muscle defects in a mouse model of human Barth syndrome. J Biol Chem.

[2] Clarke SL, Bowron A, Gonzalez IL, Groves SJ, Newbury-Ecob R, Clayton N, Martin RP, Tsai-Goodman B, Garratt V, Ashworth M (2013). Barth syndrome. Orphanet J Rare Dis.

[3] Huang Y, Powers C, Madala SK, Greis KD, Haffey WD, Towbin JA, Purevjav E, Javadov S, Strauss AW, Khuchua Z (2015). Cardiac metabolic pathways affected in the mouse model of barth syndrome. PLoS ONE.

[4] Powers C, Huang Y, Strauss A, Khuchua Z (2013). Diminished exercise capacity and mitochondrial bc1 complex deficiency in tafazzin-knockdown mice. Front Physiol.

[5] Jang S, Lewis TS, Powers C, Khuchua Z, Baines CP, Wipf P, Javadov S (2016). Elucidating mitochondrial electron transport chain supercomplexes in the heart during ischemia-reperfusion. Antioxid Redox Signal.

[6] Jefferies JL (2013). Barth syndrome. Am J Med Genet C: Semin Med Genet.

[7] Soustek MS, Falk D, Mah C, Toth M, Schlame M, Lewin A, Byrne B (2010). Characterization of a transgenic shRNA induced murine model of tafazzin deficiency. Hum Gene Ther.

[8] Phoon CK, Acehan D, Schlame M, Stokes DL, Edelman-Novemsky I, Yu D, Xu Y, Viswanathan N, Ren M (2012). Tafazzin knockdown in mice leads to a developmental cardiomyopathy with early diastolic dysfunction preceding myocardial noncompaction. J Am Heart Assoc.

[9] Hanke SP, Gardner AB, Lombardi JP, Manning PB, Nelson DP, Towbin JA, Jefferies JL, Lorts A (2012). Left ventricular noncompaction cardiomyopathy in Barth syndrome: an example of an undulating cardiac phenotype necessitating mechanical circulatory support as a bridge to transplantation. Pediatr Cardiol.

[10] Thompson WR, DeCroes B, McClellan R, Rubens J, Vaz FM, Kristaponis K, Avramopoulos D, Vernon HJ (2016). New targets for monitoring and therapy in Barth syndrome. Genet Med.

[11] Steward CG, Newbury-Ecob RA, Hastings R, Smithson SF, Tsai-Goodman B, Quarrell OW, Kulik W, Wanders R, Pennock M, Williams M (2010). Barth syndrome: an X-linked cause of fetal cardiomyopathy and stillbirth. Prenat Diagn.

[12] Neubauer S (2007). The failing heart--an engine out of fuel. N Engl J Med.

[13] Lam VH, Zhang L, Huqi A, Fukushima A, Tanner BA, Onay-Besikci A, Keung W, Kantor PF, Jaswal JS, Rebeyka IM (2015). Activating PPARalpha prevents post-ischemic contractile dysfunction in hypertrophied neonatal hearts. Circ Res.

[14] Brigadeau F, Gele P, Wibaux M, Marquie C, Martin-Nizard F, Torpier G, Fruchart JC, Staels B, Duriez P, Lacroix D. The PPARalpha activator fenofibrate slows down the progression of the left ventricular dysfunction in porcine tachycardia-induced cardiomyopathy. J Cardiovasc Pharmacol. 2007. doi:10.1097/FJC.0b013e3180544540. 10.1097/FJC.0b013e3180544540 17577106

[15] Tenenbaum A, Motro M, Fisman EZ, Tanne D, Boyko V, Behar S (2005). Bezafibrate for the secondary prevention of myocardial infarction in patients with metabolic syndrome. Arch Intern Med.

[16] Bonnefont JP, Bastin J, Laforet P, Aubey F, Mogenet A, Romano S, Ricquier D, Gobin-Limballe S, Vassault A, Behin A (2010). Long-term follow-up of bezafibrate treatment in patients with the myopathic form of carnitine palmitoyltransferase 2 deficiency. Clin Pharmacol Ther.

[17] Orngreen MC, Madsen KL, Preisler N, Andersen G, Vissing J, Laforet P (2014). Bezafibrate in skeletal muscle fatty acid oxidation disorders: a randomized clinical trial. Neurology.

[18] Orngreen MC, Vissing J, Laforet P (2014). No effect of bezafibrate in patients with CPTII and VLCAD deficiencies. J Inherit Metab Dis.

[19] Kiebish MA, Yang K, Liu X, Mancuso DJ, Guan S, Zhao Z, Sims HF, Cerqua R, Cade WT, Han X (2013). Dysfunctional cardiac mitochondrial bioenergetic, lipidomic, and signaling in a murine model of Barth syndrome. J Lipid Res.

[20] Andresen BS, Olpin S, Poorthuis BJ, Scholte HR, Vianey-Saban C, Wanders R, Ijlst L, Morris A, Pourfarzam M, Bartlett K (1999). Clear correlation of genotype with disease phenotype in very-long-chain acyl-CoA dehydrogenase deficiency. Am J Hum Genet.

[21] Karamanlidis G, Lee CF, Garcia-Menendez L, Kolwicz SC, Suthammarak W, Gong G, Sedensky MM, Morgan PG, Wang W, Tian R (2013). Mitochondrial complex I deficiency increases protein acetylation and accelerates heart failure. Cell Metab.

[22] den Boer ME, Wanders RJ, Morris AA, IJlst L, Heymans HS, Wijburg FA (2002). Long-chain 3-hydroxyacyl-CoA dehydrogenase deficiency: clinical presentation and follow-up of 50 patients. Pediatrics.

[23] Exil VJ, Gardner CD, Rottman JN, Sims H, Bartelds B, Khuchua Z, Sindhal R, Ni G, Strauss AW (2006). Abnormal mitochondrial bioenergetics and heart rate dysfunction in mice lacking very-long-chain acyl-CoA dehydrogenase. Am J Physiol Heart Circ Physiol.

[24] Xiong D, He H, James J, Tokunaga C, Powers C, Huang Y, Osinska H, Towbin JA, Purevjav E, Balschi JA (2014). Cardiac-specific VLCAD deficiency induces dilated cardiomyopathy and cold intolerance. Am J Physiol Heart Circ Physiol.

[25] Cheng Y, Hauton D (2008). Cold acclimation induces physiological cardiac hypertrophy and increases assimilation of triacylglycerol metabolism through lipoprotein lipase. Biochim Biophys Acta.

[26] Bakermans AJ, Dodd MS, Nicolay K, Prompers JJ, Tyler DJ, Houten SM (2013). Myocardial energy shortage and unmet anaplerotic needs in the fasted long-chain acyl-CoA dehydrogenase knockout mouse. Cardiovasc Res.

[27] Rau CD, Wang J, Avetisyan R, Romay MC, Martin L, Ren S, Wang Y, Lusis AJ (2015). Mapping genetic contributions to cardiac pathology induced by Beta-adrenergic stimulation in mice. Circ Cardiovasc Genet.

[28] Seibler J, Kleinridders A, Kuter-Luks B, Niehaves S, Bruning JC, Schwenk F (2007). Reversible gene knockdown in mice using a tight, inducible shRNA expression system. Nucleic Acids Res.

[29] Nakajima T, Tanaka N, Kanbe H, Hara A, Kamijo Y, Zhang X, Gonzalez FJ, Aoyama T (2009). Bezafibrate at clinically relevant doses decreases serum/liver triglycerides via down-regulation of sterol regulatory element-binding protein-1c in mice: a novel peroxisome proliferator-activated receptor alpha-independent mechanism. Mol Pharmacol.

[30] Johri A, Calingasan NY, Hennessey TM, Sharma A, Yang L, Wille E, Chandra A, Beal MF (2012). Pharmacologic activation of mitochondrial biogenesis exerts widespread beneficial effects in a transgenic mouse model of Huntington’s disease. Hum Mol Genet.

[31] Noe N, Dillon L, Lellek V, Diaz F, Hida A, Moraes CT, Wenz T (2013). Bezafibrate improves mitochondrial function in the CNS of a mouse model of mitochondrial encephalopathy. Mitochondrion.

[32] Dumont M, Stack C, Elipenahli C, Jainuddin S, Gerges M, Starkova N, Calingasan NY, Yang L, Tampellini D, Starkov AA (2012). Bezafibrate administration improves behavioral deficits and tau pathology in P301S mice. Hum Mol Genet.

[33] Jucker BM, Doe CP, Schnackenberg CG, Olzinski AR, Maniscalco K, Williams C, Hu TC, Lenhard SC, Costell M, Bernard R (2007). PPARdelta activation normalizes cardiac substrate metabolism and reduces right ventricular hypertrophy in congestive heart failure. J Cardiovasc Pharmacol.

[34] Viscomi C, Bottani E, Civiletto G, Cerutti R, Moggio M, Fagiolari G, Schon EA, Lamperti C, Zeviani M (2011). In vivo correction of COX deficiency by activation of the AMPK/PGC-1alpha axis. Cell Metab.

[35] Yatsuga S, Suomalainen A (2012). Effect of bezafibrate treatment on late-onset mitochondrial myopathy in mice. Hum Mol Genet.

[36] Dillon LM, Hida A, Garcia S, Prolla TA, Moraes CT (2012). Long-term bezafibrate treatment improves skin and spleen phenotypes of the mtDNA mutator mouse. PLoS ONE.

[37] Kajosaari LI, Backman JT, Neuvonen M, Laitila J, Neuvonen PJ (2004). Lack of effect of bezafibrate and fenofibrate on the pharmacokinetics and pharmacodynamics of repaglinide. Br J Clin Pharmacol.

[38] Wenz T, Diaz F, Spiegelman BM, Moraes CT (2008). RETRACTED: activation of the PPAR/PGC-1alpha pathway prevents a bioenergetic deficit and effectively improves a mitochondrial myopathy phenotype. Cell Metab.

[39] Prince PS, Sathya B (2010). Pretreatment with quercetin ameliorates lipids, lipoproteins and marker enzymes of lipid metabolism in isoproterenol treated cardiotoxic male Wistar rats. Eur J Pharmacol.

[40] Olsson AG, Lang PD. Dose-response study of bezafibrate on serum lipoprotein concentrations in hyperlipoproteinanemia. Atherosclerosis. 1978;(4):421–8. 10.1016/0021-9150(78)90137-5 215174

